# PARTICIPATION IN AND EXCLUSION FROM EXTENDED LATE WORKING LIFE DURING THE DEMOGRAPHIC, DIGITAL, AND GREEN TRANSITION

**DOI:** 10.1093/geroni/igad104.1786

**Published:** 2023-12-21

**Authors:** Andreas Motel-Klingebiel

## Abstract

Policies aim to extend working lives by investing in the capacity and employability of older workers, changing the regulatory framework, promoting innovative life-course policies, and advancing lifelong learning, which gains relevance under the impact of increasingly aging populations, digitalisation, and the green transition. However, as some national, branch, and company policies are counterproductive, encouraging early exit and perpetuating ageist training and recruitment practices, working longer may not be equally achievable and beneficial for all workers. This symposium presents research on exclusion and inequality in late working life, providing evidence for policy innovation towards inclusive extended work and sustainable working conditions. It combines comparative multi-level research based on registry and survey data as well as qualitative information. Contributions address the opportunities and limitations of equal and inclusive extended work under the impact of population aging, digitalisation, and the green transition. This includes a conceptual introduction to research and policy on extended late work in Europe during the triple demographic, digital and green transition, an analysis of age discrimination in company and branch policies based on expert interview data, an examination of the perceived significance of lifelong learning for exclusion risks, an analysis of risk experiences associated with extended work, and a commentary discussing the findings in the context of gerontological debates on exclusion and precarity in later life. The (co-)authors represent a mix of early career, mid-career, and senior scholars with interdisciplinary backgrounds from Europe and North America. The audience is invited to discuss concepts, analyses, and conclusions with the presenters.

